# Identification of an amino acid metabolism-associated gene signature predicting the prognosis and immune therapy response of clear cell renal cell carcinoma

**DOI:** 10.3389/fonc.2022.970208

**Published:** 2022-09-08

**Authors:** Fan Zhang, Junyu Lin, Daiwen Zhu, Yongquan Tang, Yiping Lu, Zhihong Liu, Xianding Wang

**Affiliations:** ^1^ Department of Urology, Institute of Urology, West China Hospital, Sichuan University, Chengdu, China; ^2^ West China Clinical Medical College, West China Hospital, Sichuan University, Chengdu, China; ^3^ Department of Pediatric Urology, West China Hospital, Sichuan University, Chengdu, China

**Keywords:** ccRCC, amino acid metabolism, signature, anti-PD-1 therapy, prognosis

## Abstract

**Background:**

The upregulation of amino acid metabolism is an essential form of metabolic reprogramming in cancer. Here, we developed an amino acid metabolism signature to predict prognosis and anti-PD-1 therapy response in clear cell renal cell carcinoma (ccRCC).

**Methods:**

According to the amino acid metabolism-associated gene sets contained in the Molecular Signature Database, consensus clustering was performed to divide patients into two clusters. An amino acid metabolism-associated signature was identified and verified. Immune cell infiltrates and their corresponding signature risk scores were investigated. Two independent cohorts of clinical trials were analyzed to explore the correspondence between the signature risk score and the immune therapy response.

**Results:**

Two clusters with different amino acid metabolic levels were identified by consensus clustering. The patients in the two clusters differed in overall survival, progression-free survival, amino acid metabolic status, and tumor microenvironment. We identified a signature containing eight amino acid metabolism-associated genes that could accurately predict the prognosis of patients with ccRCC. The signature risk score was positively correlated with infiltration of M1 macrophages, CD8+ T cells, and regulatory T cells, whereas it was negatively correlated with infiltration of neutrophils, NK cells, and CD4+ T cells. Patients with lower risk scores had better overall survival but worse responses to nivolumab.

**Conclusion:**

Amino acid metabolic status is closely correlated with tumor microenvironment, response to checkpoint blockade therapy, and prognosis in patients with ccRCC. The established amino acid metabolism-associated gene signature can predict both survival and anti-PD-1 therapy response in patients with ccRCC.

## Introduction

Renal cell carcinoma (RCC) refers to cancer that originates from the renal epithelium. As one of the most common cancers worldwide, RCC accounts for more than 140,000 cancer-related deaths annually (1). RCC has a higher incidence in developed countries and in men (2). It encompasses more than 10 subtypes, among which the most common is clear-cell renal cell carcinoma (ccRCC), contributing to the majority of kidney cancer–related deaths (3). However, robust predictive biomarkers for prognosis and rational treatment choices for ccRCC are lacking.

Increasing evidence has indicated a role for metabolic reprogramming in many types of cancers (4), including ccRCC (5). In addition to diverting glucose metabolism, amino acid metabolic reprogramming is important in cancer development. Amino acids are not only essential nutritional substrates and sources of energy for tumor cells but also associated with the metabolism of glucose, lipids, and nucleotides, making them vital for tumor proliferation, invasion, and metastasis. Normal cells have lower amino acid requirements than tumor cells; this difference contributes to the metabolic vulnerability of malignant cells and provides a principle for amino acid depletion therapy (6). However, the efficacy of amino acid depletion therapy is highly dependent on the tumor microenvironment (7). Therefore, the amino acid status of tumor cells may correlate with immune cell infiltrates and has great potential in tumor therapy (6).

Several studies have found a close correlation between the expression of amino acid metabolism-associated genes and various types of cancers, including gliomas (8), breast cancer (9), and hepatocellular carcinoma (10), supporting the importance of amino acid metabolism in cancer. In ccRCC, previous studies found that except for glucose and fatty acid metabolism, the metabolism of amino acids, including tryptophan, ornithine, arginine, citrulline, and glutamine, is also reprogrammed (5). However, an amino acid metabolism-associated gene set has not been systematically studied in ccRCC.

Therefore, in the current study, we aimed to conduct systematic and comprehensive research on amino acid metabolic features in ccRCC and establish a reliable prognostic model to predict the clinical outcomes of ccRCC and help make appropriate therapeutic decisions.

## Methods

### Dataset collection

The clinical, pathological, and transcriptome data of patients with ccRCC were obtained from The Cancer Genome Atlas (TCGA) data portal (https://portal.gdc.cancer.gov/). Additionally, according to the cBioPortal online database (http://www.cbioportal.org/), a validation cohort (n=446) was established and confirmed the results (11).

### Consensus clustering and enrichment analysis

Three amino acid metabolism-associated gene sets (amino acid metabolic process, modified amino acid metabolic process, and amino acid and derivative metabolic process) were obtained from the Molecular Signature Database (http://www.broad.mit.edu/gsea/msigdb/) (12). After removing overlapping genes, we retrieved an amino acid metabolism-associated gene set of 460 genes (**Supplementary Material 1**). We conducted a consensus clustering algorithm using the R package “ConsensusClusterPlus” (13). The most differentially expressed genes (DEGs) between the two clusters were visualized by the “pheatmap” R package. Moreover, to better understand the underlying functions of the potential targets, the R package “ClusterProfiler” was utilized for enrichment analysis. Using the R packages “survival,” survival curves were generated. Using chi-square tests or Fisher’s exact tests for categorical variables according to the theoretical frequency and Student’s *t*-tests for continuous variables, clinical and pathological characteristics were compared.

### Gene signature identification

To identify the key amino acid metabolic genes associated with ccRCC, we first screened the genes correlated with tumor prognosis using univariate COX methods. Then, using the “glmnet” R package, the least absolute shrinkage and selection operator (LASSO) Cox regression algorithm was performed and finally followed by multivariate COX proportional hazards regression analysis, resulting in an identification of eight genes. A nomogram was then established through the “rms” and “survival” R packages. Furthermore, we verified the signature using the data from the cBioPortal datasets (14). The web server GEPIA was employed to analyze the Kaplan–Meier curves of the eight signature genes (15).

### Amino acid signature predicted tumor response to nivolumab

RNA-seq and survival data were obtained and analyzed from previous clinical trials, including NCT01668784 (a phase III clinical study comparing everolimus and nivolumab in patients with previously treated metastatic ccRCC, CheckMate025) and NCT01354431 (a phase II study of nivolumab in patients with metastatic ccRCC, CheckMate 010) (16). Based on tumor shrinkage after therapy, patients were divided into clinical benefit (CB), intermediate clinical benefit (ICB), and no clinical benefit (NCB) groups. Subsequent analyses of patients with ICB in the CB group were conducted. We investigated the CB/NCB ratio in patients with high- or low-risk score and the prognosis of patients who underwent anti-PD-1 therapy (nivolumab).

## Results

### Patient information collection and consensus clustering

We established a cohort of 530 patients according to TCGA-ccRCC data, which included RNA sequencing data and clinical and pathological information, for the following analysis. Three amino acid metabolism-associated gene sets were retrieved, and 460 genes were identified after removing the overlapping genes (**Supplementary Material 1**).

**Figure 1:**
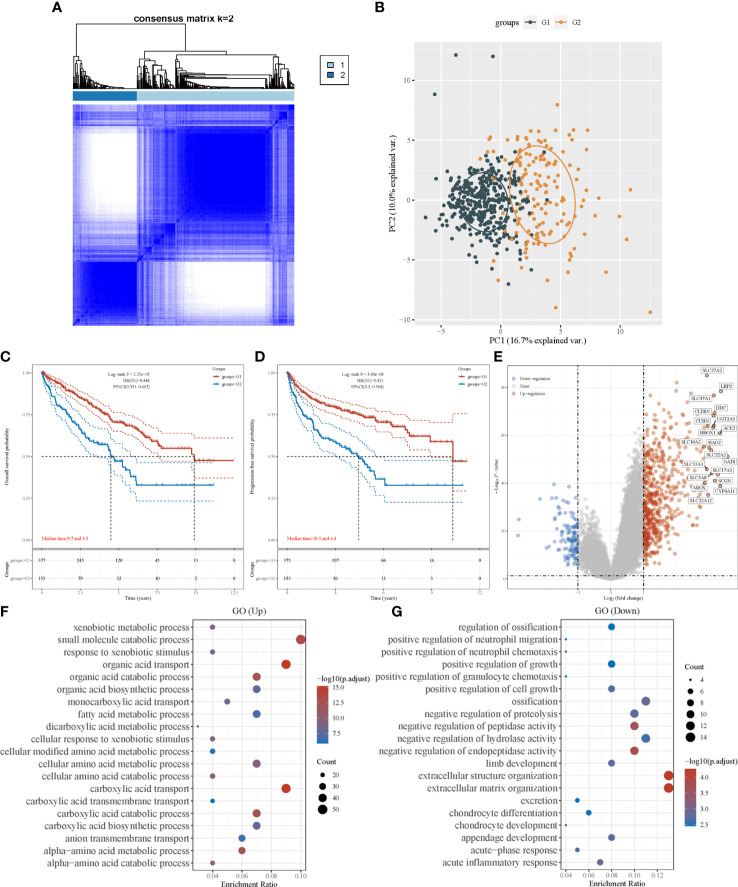
Consensus clustering analysis to identify the genomic subtype of ccRCC based on an amino acid metabolism-associated gene set. **(A)** Consensus clustering matrix of 530 samples from TCGA dataset for k=2. **(B)** PCA distinguishing patients’ risk groups. Kaplan–Meier curve of overall survival **(C)** and progression free survival **(D)** of patients with ccRCC in two clusters. **(E)** Volcano plot of DEGs between two clusters with different metabolic statuses. The red and blue points represent up- and downregulated genes with statistical significance, respectively. **(F)** GO analysis of DEGs that were upregulated in cluster 1. **(G)** GO analysis of DEGs that were downregulated in cluster 1. PCA, principal component analysis; DEG, differentially expressed genes.

A consensus clustering algorithm was utilized to classify patients with ccRCC into clusters with isolated amino acid metabolic status. Two different clusters (cluster 1 and cluster 2) were identified using the optimal grouping method, according to the empirical cumulative distribution function (CDF) plot (**Supplementary Material 2**), consensus clustering matrix (**Figure 1A**; **Supplementary Material 3**), and principal component analysis (**Figure 1B**). The overall survival (OS) of patients from cluster 1 was significantly longer (hazard ratio [HR]: 0.448, 95% confidence interval [CI]:0.331–0.607, P<0.001, **Figure 1C**), and similar better progression-free survival (PFS) was also observed in cluster 1 (HR: 0.441, 95% CI: 0.3–0.564, P<0.001, **Figure 1D**). A heatmap of amino acid metabolic gene expression between the two clusters is shown in **Supplementary Material 4A**, indicating that the two clusters were significantly different in amino acid metabolic status.

To investigate the differences between the clusters, their clinical and pathological characteristics were explored (**Table 1**). The two clusters differed in survival status (P<0.001), sex (P=0.025), lymph node metastasis (P=0.007), tumor stage (P<0.001), and histological grade (P<0.001), implying that amino acid metabolism may correlate with these clinicopathological features.

**Table 1:** Clinicopathological feathers between the two clusters.

Characteristics	Cluster 1	Cluster2	P value
Survival status			<0.001
Alive	276	81	
Dead	101	72	
Sex			0.025
Female	144	42	
Male	233	111	
Race			0.519
Asian	6	2	
Black	36	20	
White	328	131	
Lymph node metastasis			0.007
N0	168	71	
N1	6	10	
NX	203	72	
Metastasis			<0.001
M0	329	111	
M1	43	37	
MX	5	5	
Tumor stage			<0.001
I	211	54	
II	40	17	
III	81	42	
IV	44	38	
Pathological grade			<0.001
G1	13	1	
G2	179	48	
G3	153	53	
G4	29	46	
GX	0	5	

### Enrichment analysis

We identified DEGs in the two clusters and conducted enrichment analysis to investigate the underlying mechanism and pathway difference-correlated amino acid metabolism. The top DEGs between the two clusters were analyzed using a heatmap (**Supplementary Material 4B**). The most upregulated genes were *SLC27A2*, *LRP2*, and *DDC*, whereas the most downregulated genes were *TRNP1*, *TMEM158*, and *BDKRB1* (**Figure 1E**).

Setting the adjusted P-value and fold-change threshold at 0.05 and 1.5, we then performed Gene Ontology (GO)/Kyoto Encyclopedia of Genes and Genomes (KEGG) analysis. Signaling pathway enrichment verified the different amino acid metabolism statuses between the two clusters, and the amino acid metabolism of cluster 1 was activated (**Supplementary Material 5A**). GO enrichment analysis showed similar results, with upregulated genes in cluster 1 being strongly associated with amino acid metabolism. Other relevant signaling pathways were mainly enriched in carboxylic acid transport, organic acid transport, and small-molecule catabolic processes (**Figure 1F**). DEGs upregulated in cluster 2 were majorly enriched in extracellular structure organization, negative regulation of hydrolase activity, and several immune terms, including positive regulation of granulocyte chemotaxis, positive regulation of neutrophil migration, positive regulation of neutrophil chemotaxis, and acute inflammatory response (**Figure 1G**). In the KEGG pathway analysis, we found that the DEGs were mainly enriched in human papillomavirus infection, cytokine–cytokine receptor interactions, and immune-associated pathways, which involve the IL-17 signaling pathway and phagosomes (**Supplementary Material 5B**). These results revealed that the activation of amino acid metabolic processes was a feature of cluster 1, whereas the upregulation of tumor-related immunogenicity may be an important characteristic of cluster 2.

Next, drug sensitivity between the two clusters was estimated. Patients with different amino acid metabolic status also exhibited different IC_50_ scores of pazopanib, sunitinib, and sorafenib (**Supplementary Material 6**) for ccRCC, implying that amino acid metabolism may affect the efficacy of these targeted drugs. However, these conclusions require further verification with clinical drug trials.

### Signature development

**Figure 2:**
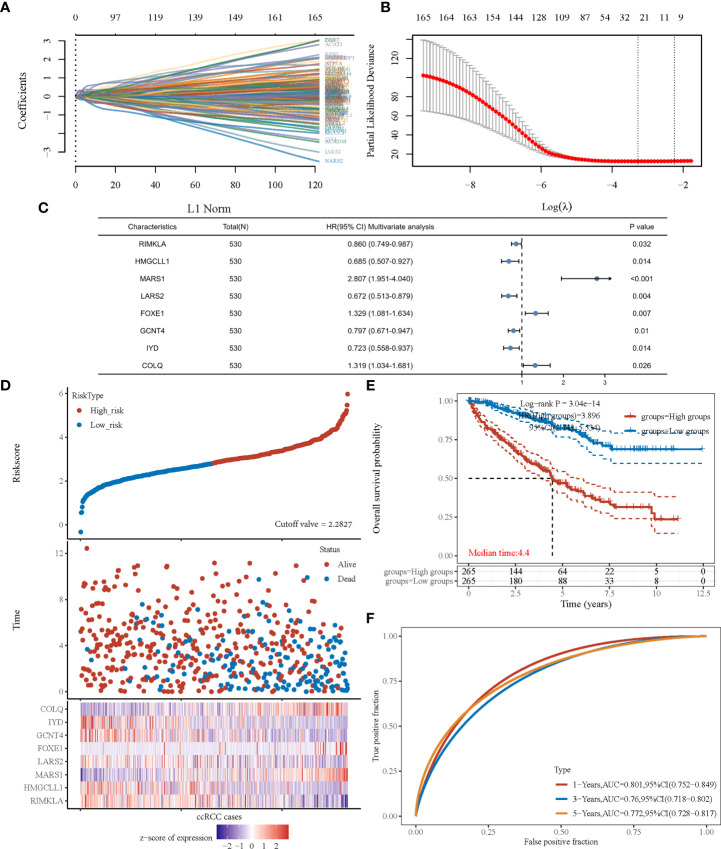
The prognostic signature was established based on eight prognostic amino acid metabolism-associated genes. **(A)** LASSO coefficient profiles of the genes associated with the amino acid metabolism of ccRCC. **(B)** Partial likelihood deviance is plotted versus log (λ). **(C)** Forest plot of multivariate COX analysis of eight genes involved in the signature. **(D)** The risk score of each sample based on the amino acid metabolism-associated signature. Patients were divided into low- and high-risk groups according to the median value of the risk score. The high/low expression of eight genes that were involved in the prognostic signature are shown in red/blue in each sample. **(E)** Kaplan–Meier curve of overall survival differences stratified by signature risk score. **(F)** The ROC curves of the signature for overall survival at 1, 3, and 5 years. LASSO, least absolute shrinkage and selection operator; ROC, receiver operating characteristic.

After revealing that amino acid metabolism was correlated with prognosis of patients with ccRCC, an amino acid metabolism-associated gene signature was developed to identify key amino acid metabolic genes and better predict prognosis. Using the univariate Cox regression method, we identified 166 of the 460 genes that were significantly correlated with ccRCC prognosis (P<0.001). The LASSO algorithm was used, and 25 amino acid metabolism-correlated genes were identified, namely *ASNS*, *CARS1*, *SLC7A5*, *ACADSB*, *DMGDH*, *GFPT2*, *CRAT*, *PYCR1*, *RIMKLA*, *HMGCLL1*, *BCKDHA*, *MARS1*, *LARS2*, *ILVBL*, *SARS2*, *ACADL*, *FOXE1*, *CARS2*, *GCNT4*, *IYD*, *COLQ*, *MCCC2*, *VNN3*, *DPEP1*, and *NOS3* (**Figure 2A, B**). Finally, the Cox coefficient was calculated using a multivariate Cox regression analysis (**Figure 2C**). We obtained the following signature with eight amino acid metabolic genes: risk score= (-0.151 × *RIMKLA* level) + (-0.378× *HMGCLL1* level) + (1.032× *MARS1* level) + (-0.398× *LARS2* level) + (0.284× *FOXE1* level) + (-0.227× *GCNT4* level) + (-0.324× *IYD* level) + (0.277× *COLQ* level).

**Figure 3:**
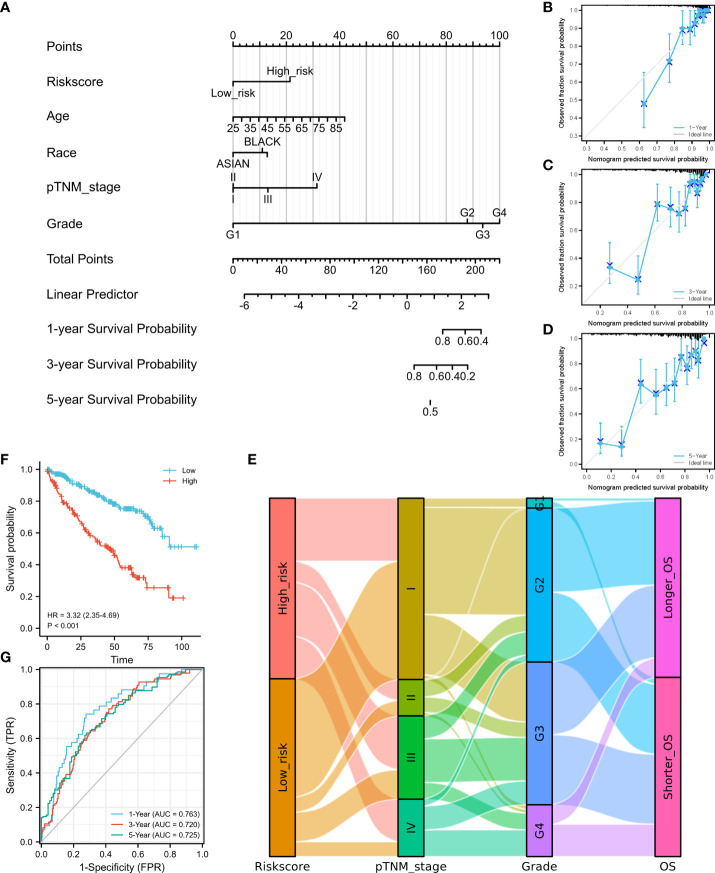
Construction of a nomogram and independent signature validation. **(A)** Nomogram for predicting 1-, 3-, or 5-year OS in patients with ccRCC. **(B)** The calibration plots for predicting 1-year OS. **(C)** The calibration plots for predicting 3-year OS. **(D)** The calibration plots for predicting 5-year OS. **E** Sankey diagram showing the association between signature risk scores and clinicopathological characteristics. **(F)** Validation of the signature in overall survival based on data from the cBioPortal online database. **(G)** The ROC curves of the signature validation for overall survival at 1, 3, and 5 years. OS, overall survival; ROC, receiver operating characteristic.

According to the risk score calculated using the developed signature, patients with ccRCC could be classified as high or low risk (**Figure 2D**). Survival curves showed that the OS was significantly different between patients with high- and low-risk scores (**Figure 2E**, P<0.001), with 1-, 3-, and 5-year OS AUCs of 0.801, 0.760, and 0.772, respectively (**Figure 2F**). Moreover, we used an independent cBioPortal cohort as a validation set to validate the predictive value of the amino acid metabolic signature. Risk score-based survival curves showed similar trends, and a significantly worse OS was observed in the high-risk group than in the low-risk group (**Figure 3F**, P<0.001). In the validation cohort, the AUCs for the 1-, 3-, and 5-year OS were 0.763, 0.720, and 0.725, respectively (**Figure 3G**).

We then established a nomogram containing the available clinical and pathological characteristics and calculated the signature risk score (**Figure 3A**). The accuracy of this nomogram was verified using calibration curves of the 1- (**Figure 3B**), 3- (**Figure 3C**), and 5-year survival rates (**Figure 3D**). The correlation between signature risk scores and clinicopathological characteristics was determined using a Sankey diagram (**Figure 3E**). Multivariate Cox regression analyses were also conducted, and the predictive signature served as an independent prognostic risk factor (HR: 2.581; 95% CI: 1.786–3.732, P<0.001).

### High-risk patients are correlated with increased response to anti-PD-1 immunotherapy

**Figure 4:**
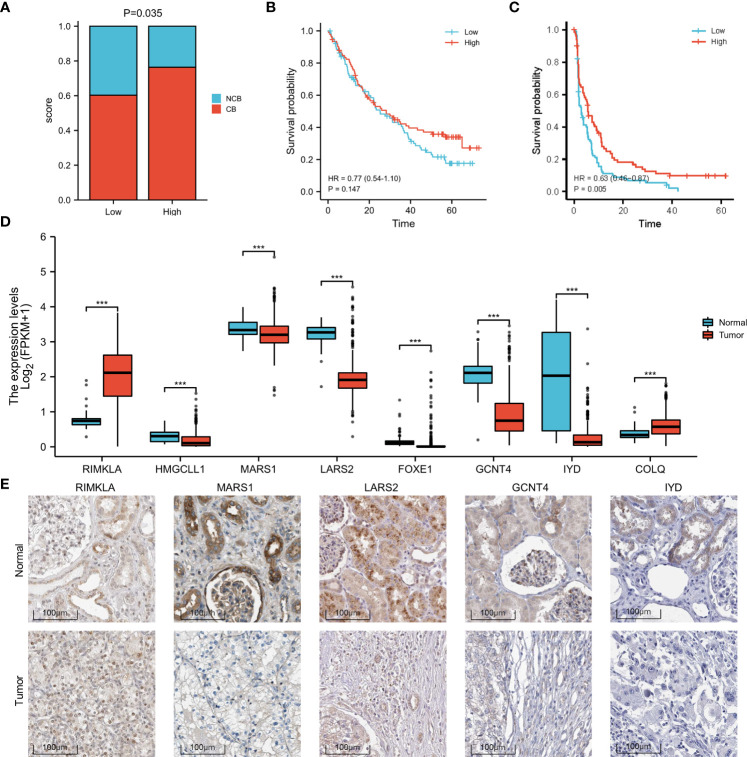
Increased response to anti-PD-1 immunotherapy in high-risk patients with ccRCC and differences in the expression of the signature genes. **(A)** Proportion of CB versus NCB in patients with high- or low-risk scores. **(B)** OS in high- and low-risk patients who underwent anti-PD-1 immunotherapy. **(C)** PFS in high- and low-risk patients who underwent anti-PD-1 immunotherapy. **(D)** Box plot of the difference in expression of signature genes between normal and ccRCC tissues, according to TCGA sequencing data. **(E)** The expression difference of *RIMKLA*, *MARS1*, *LARS2*, *GCNT4*, and *IYD* between tumor and normal tissues at the protein level, according to Human Protein Atlas (HPA) cohort. OS, overall survival; PFS, progress free survival. ***p < 0.001.

**Figure 5:**
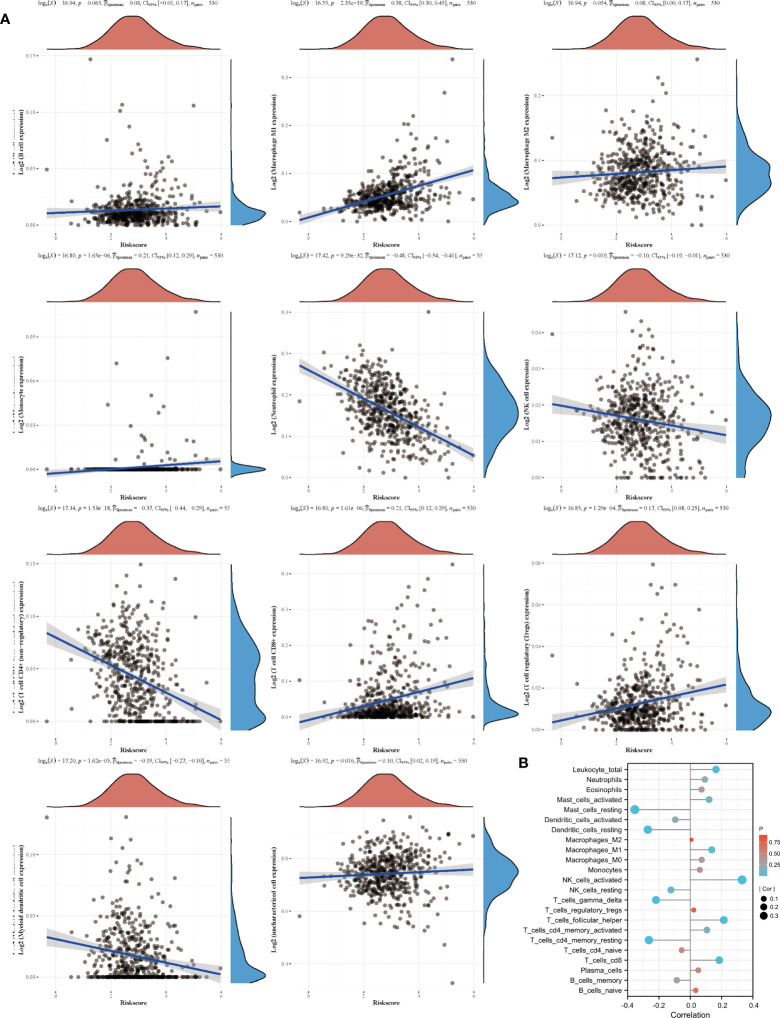
Signature and immune cell infiltrates. **(A)** Correlation of immune cell infiltrates with risk score according to TCGA datasets. **(B)** Immune cell infiltration in patients receiving nivolumab therapy of two clinical trials.

Based on the sequencing and prognostic data of 156 patients with advanced ccRCC, we found that patients with a higher risk score of the amino acid metabolic signature had a higher proportion of CB to anti-PD-1 immunotherapy (nivolumab) than patients with lower risk scores (**Figure 4A**, P=0.035). We found that patients in the high-risk score group tended to have a worse OS and PFS. However, when they received anti-PD-1 immunotherapy, the high-risk score group had a similar OS (HR: 0.77, 95% CI: 0.54–1.10, P=0.147, **Figure 4B**) and longer PFS (HR: 0.63, 95% CI: 0.46–0.87, P=0.005, **Figure 4C**) compared with the low-risk score group. We also found a strong correlation between the signature risk score and immune microenvironment according to TCGA datasets (**Figure 5A**) and data from clinical trials of 156 patients with advanced ccRCC (**Figure 5B**). The signature risk score was positively correlated with the infiltration of M1 macrophages, CD8+ T cells, and T-cell regulatory cells, whereas it was negatively correlated with neutrophils, NK cells, and T-cell CD4+ cells based on TCGA data. In the 156 patients with advanced ccRCC, the signature risk score was significantly correlated with the infiltration of CD8+ T cells (Cor: 0.185, P=0.013), resting memory of CD4+ T cells (Cor: –0.264, P<0.001), follicular helper T cells (Cor: 0.214, P=0.004), gamma delta T cells (Cor: 0.219, P=0.003), activated NK cells (Cor: 0.331, P<0.001), resting dendritic cells (Cor: –0.271, P<0.001), resting mast cells (Cor: –0.354, P<0.001), and leukocytes (Cor: 0.164, P=0.028).

### Differences in gene expression

The different expression patterns between normal and tumor tissues of the eight genes involved in the signature were explored at the mRNA (**Figure 4D**) and protein levels (**Figure 4E**). Based on the Human Protein Atlas (HPA) cohort, among the eight amino acid metabolism-associated genes, we found that the expression of *RIMKLA*, *MARS1*, *LARS2*, *GCNT4*, and *IYD* was different between normal tissue and ccRCC at the protein level. Survival analysis of the eight genes was performed (**Supplementary Material 7**).

## Discussion

Metabolic reprogramming is a hallmark of cancer (17). In addition to the well-described Warburg effect, namely enhanced glycolysis (18), the regulation of amino acid metabolism is another crucial reprogrammed metabolic pathway in cancer (4). For example, glutamine upregulation is crucial for cancer cells. Glutamine is not only an essential nutritional resource for cancer cells but also provides nitrogen atoms to synthesize hexosamines, nucleotides, and amino acids, promoting the survival and growth of cancer cells (19). Another example is the expression of asparagine synthetase, which is correlated with worse prognosis in glioma and neuroblastoma, mainly because of the increasing need for protein synthesis in rapidly proliferating cancer cells (20). L-Asparaginase has been used in cancer therapy (21). Therefore, the selective inhibition of amino acid metabolism has shown promising potential in cancer therapy (7).

In ccRCC, in addition to the well-established Warburg effect, a glutamine-dependent pathway, namely reductive carboxylation, has been observed (22). Additionally, the metabolism of tryptophan and arginine has been reprogrammed in ccRCC (5). In the current study, according to TCGA and cBioPortal sequencing and clinicopathological data, we revealed, for the first time, that the expression of amino acid metabolism-associated genes was significantly associated with the progression and prognosis of ccRCC, similar to findings in other types of cancer, including gliomas, breast cancer, and hepatocellular carcinoma, as reported previously (8–10). Our findings confirm the vital role of amino acid metabolism in ccRCC.

GO/KEGG enrichment analysis demonstrated that amino acid metabolism may have an impact on immune and inflammatory responses, suggesting a crosstalk between the amino acid metabolic status and the tumor immune microenvironment. Emerging evidence suggests that amino acid metabolism is associated with immune regulation in patients with ccRCC. For instance, a previous study found that the level of tryptophan is decreased in ccRCC, while the levels of kynurenine and quinolinate are increased, indicating an enhanced utilization of tryptophan (23). Considering the immunosuppressive effects of kynurenine and quinolinate, the regulation of tryptophan leads to the suppression of the immune system and facilitates cancer growth. Further analyses revealed that the signature risk score was significantly correlated with CD8+ T-cell infiltration. A high level of tumor CD8+ T-cell infiltration reportedly correlates with worse prognosis in patients with ccRCC (24), possibly explaining the worse prognosis observed in patients with higher risk scores.

ccRCC is one of the most highly immune-infiltrated solid tumors (25). Rich leukocyte infiltration is a key characteristic of ccRCC, including CD8+ T, CD4+ T, NK, and myeloid cells with characteristics of macrophages and neutrophils (26). Despite robust T-cell infiltration, ccRCC continues to progress. In addition, high T-cell levels were not associated with a good prognosis for ccRCC. One rational explanation for this phenomenon is that many T cells are immunosuppressed phenotypes (27).

Another important finding of the current study was that the amino acid metabolism-associated gene signature can also predict the response to checkpoint blockade therapy; specifically, patients with a higher risk score for the amino acid metabolic signature had a better response to anti-PD-1 immunotherapy, except for an increased infiltration of CD8+ T cells. The response to checkpoint blockade therapy has been widely reported to correlate with microenvironment features, especially increased T-cell infiltration (28). A previous study demonstrated that sufficient T-cell infiltration in tumor tissues is a prerequisite for the response to PD-L1 blockade (29). A previous study on ccRCC found that the interplay between somatic alterations and immune infiltration could regulate the response to PD-1 blockade therapy (16). This finding further confirms the observed close correlation between amino acid metabolism and immune regulation. Although checkpoint blockade therapy is beneficial for many patients with ccRCC (30), a large proportion of patients still respond poorly to checkpoint blockade therapy (31). To date, reliable biomarkers for responses to checkpoint blockade in patients with ccRCC are lacking. Therefore, the signature established in the current study offers a potential tool for predicting the patient response to checkpoint blockade therapy.

Owing to the strong correlation between the amino acid metabolic status and clinical and pathological characteristics in patients with ccRCC, we developed an amino acid metabolism-associated signature to identify high- or low-risk groups with different amino acid metabolic status. In the current study, a signature involving eight genes showed potent efficiency in predicting prognosis. Among these eight genes, we found that the expression of *RIMKLA*, *MARS1*, *LARS2*, *GCNT4*, and *IYD* was significantly different between ccRCC and normal tissues. *RIMKLA*, the ribosomal modification protein rimK-like family member A gene, encodes an *N*-acetylaspartylglutamate synthetase that synthesizes the N-acetylated tripeptide *N*-acetylaspartylglutamylglutamate *(* 32). *MARS1* encodes methionyl-transfer RNA synthetase 1, which is an essential translation factor. Pathogenic mutations in *MARS1* can cause trichothiodystrophy, a rare hereditary neurodevelopmental disorder characterized by sulfur-deficient brittle hair, nails, and scaly skin (33). High expression of methionyl-transfer RNA synthetase reportedly has a poor prognosis in breast cancer (34). *LARS2* encodes mitochondrial leucyl-transfer RNA synthetase, which attaches leucine to its cognate tRNA. Biallelic pathogenic variants of *LARS2* can cause a wide phenotypic spectrum, including deafness, ovarian failure, leukodystrophy, and lactic acidosis due to mitochondrial function impairment (35, 36). Variants in *LARS2* have also been found to correlate with the risk of type 2 diabetes (37). Previous studies have indicated that *LARS2* plays a role in various types of cancer, including non-small cell lung cancer (38), gastric cancer (39), and acute myelocytic leukemia (40). *GCNT4* encodes subunit 4 of glucosaminyl *N*-acetyl transferase. Previous studies have reported decreased expression of *GCNT4* in gastric cancer tissues (41). Further studies have revealed that overexpression of *GCNT4* can prevent the growth of gastric cancer cells by regulating the TGF-β1/SMAD3 pathway (42). *IYD* encodes iodotyrosine deiodinase, which reportedly has a suppressive effect on hepatocellular carcinoma cells by inhibiting cell glycolysis (43). Further studies are needed to determine the exact mechanisms by which these genes affect ccRCC.

The current study had some limitations. First, few patients with higher tumor grades and pathologic stages were included in the clinical trial cohort, which may have resulted in the limited prognostic value of the signature in these patients. Second, we conducted most analyses at the transcriptomic level, and further protein-level analysis that is crucial needs to be conducted. Third, our findings were mainly based on data from the cBioPortal and TCGA datasets. Other independent cohorts having great value need to be validated.

In conclusion, our study revealed that the amino acid metabolic status is closely correlated with the immune microenvironment, response to checkpoint blockade therapy, and prognosis of patients with ccRCC. The amino acid metabolism-associated gene signature established in the current study can be used to predict not only the survival of patients with ccRCC but also the potential response to checkpoint blockade therapy, which can benefit patients in making an appropriate management choice.

## Data availability statement

The original contributions presented in the study are included in the article/**Supplementary Material**. Further inquiries can be directed to the corresponding authors.

## Author contributions

FZ and JL wrote the main manuscript text. Study was designed by XW and ZL. Data analysis were done by FZ, JL and DZ. FZ and JL prepared figures and tables. All authors contributed to the article and approved the submitted version.

## Funding

This work was supported by grants from the Sichuan Science and Technology Program [grant number 2019YJ0133, 2021YFS0071]; Chengdu Science and Technology Program [grant number 2019-YF05-00084-SN]; The funders had no role in study design, data collection or analysis, preparation of the manuscript, or the decision to publish.

## Conflict of interest

The authors declare that the research was conducted in the absence of any commercial or financial relationships that could be construed as a potential conflict of interest.

## Publisher’s note

All claims expressed in this article are solely those of the authors and do not necessarily represent those of their affiliated organizations, or those of the publisher, the editors and the reviewers. Any product that may be evaluated in this article, or claim that may be made by its manufacturer, is not guaranteed or endorsed by the publisher.

## Supplementary material

Supplementary material 1 An amino acid metabolism-associated gene set containing 460 genes.

Supplementary material 2 CDF plot of consensus clustering for k = 2 to k = 6. CDF: Cumulative distribution function.

Supplementary material 3 Consensus clustering matrix of 530 samples from TCGA dataset for k =3 **(A)**, 4 **(B)**, 5 **(C)**, and 6 **(D)**.

Supplementary material 4
**(A)** Heatmap of amino acid metabolism-associated gene expression in different clusters. Red represents high gene expression, and blue represents low expression. **(B)** Heatmap of the top 50 up- and downregulated genes with the most differential changes in two clusters.

Supplementary material 5 KEGG analysis of DEGs that were up- **(A)** and down- **(B)** regulated in cluster 1. KEGG: Kyoto Encyclopedia of Genes and Genomes. DEG: differentially expressed genes.

Supplementary material 6 Chemotherapy resistance to pazopanib, sunitinib and sorafenib of the two clusters. *p < 0.05, **p < 0.01, ***p < 0.001.

Supplementary material 7 Survival curves of eight genes involved in the prognostic signature.
