# Supplementary figures and images for: Identification of an amino acid metabolism-associated gene signature predicting the prognosis and immune therapy response of clear cell renal cell carcinoma
Source: Front Oncol. 2022 Sep 8;12:970208. doi: 10.3389/fonc.2022.970208 (PMC9493051; doi:10.3389/fonc.2022.970208)

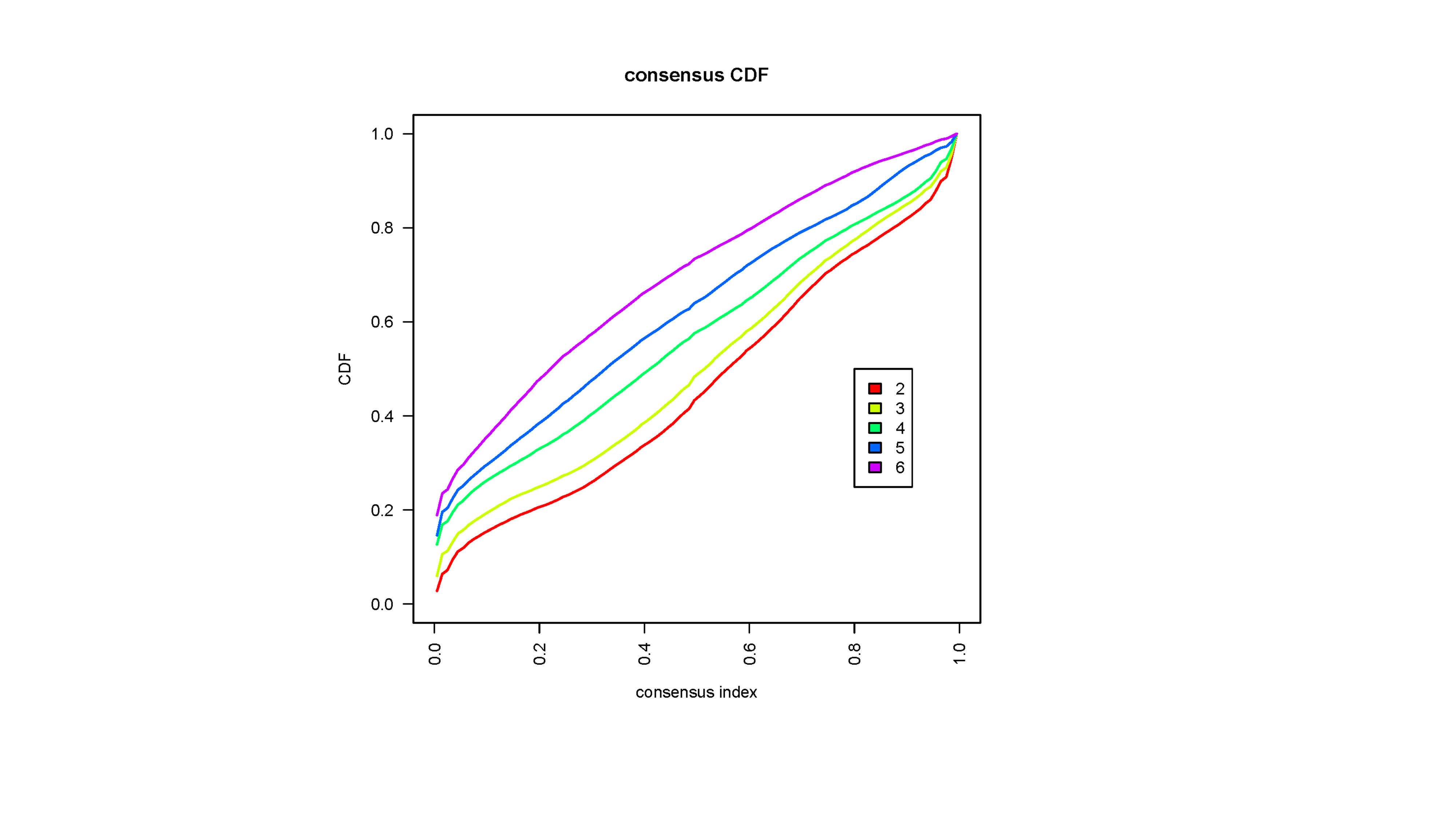

Supplement: Supplementary material 1 — An amino acid metabolism-associated gene set containing 460 genes. [file Image_1.tif]

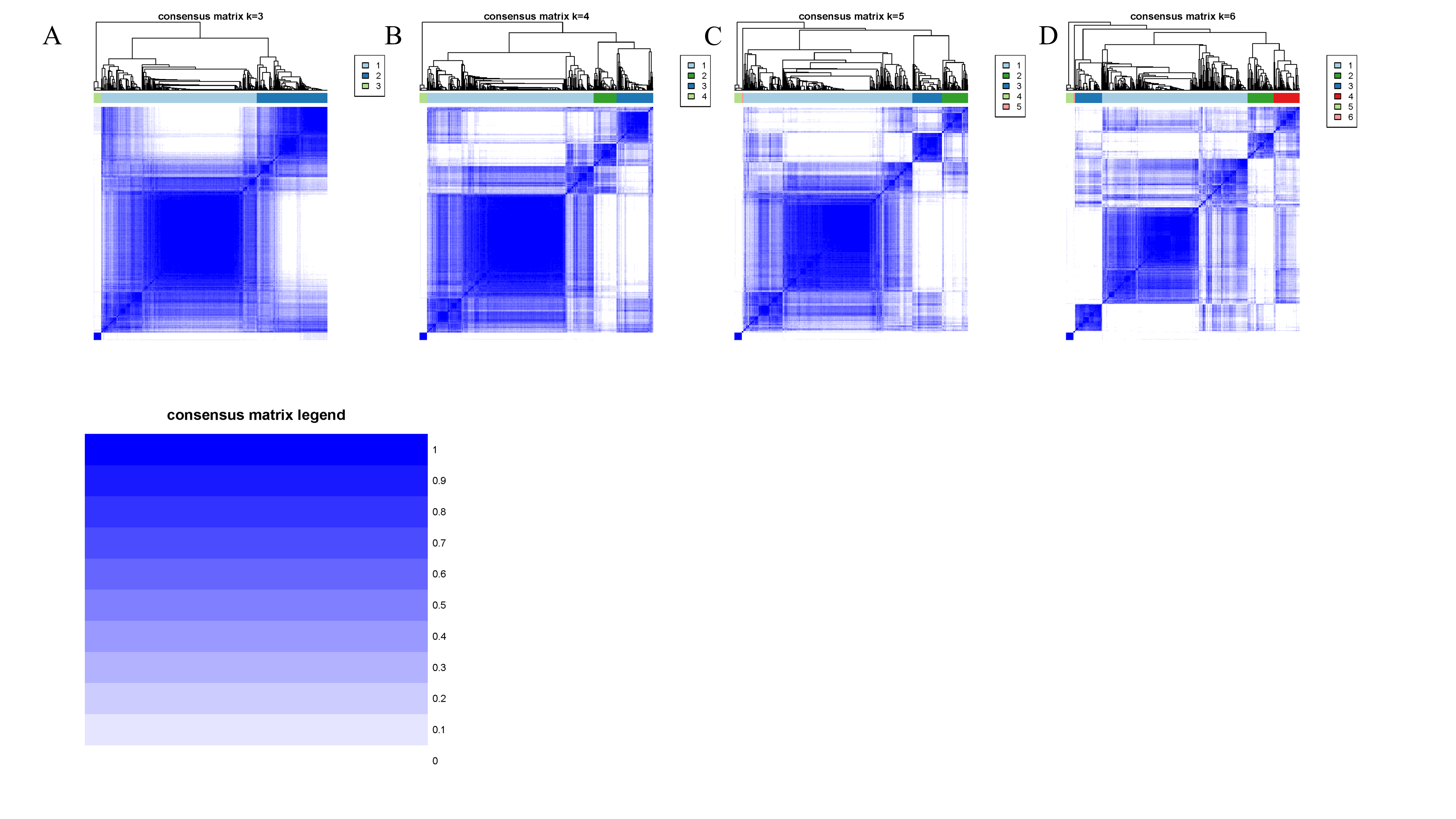

Supplement: Supplementary material 2 — CDF plot of consensus clustering for k = 2 to k = 6. CDF: Cumulative distribution function. [file Image_2.tif]

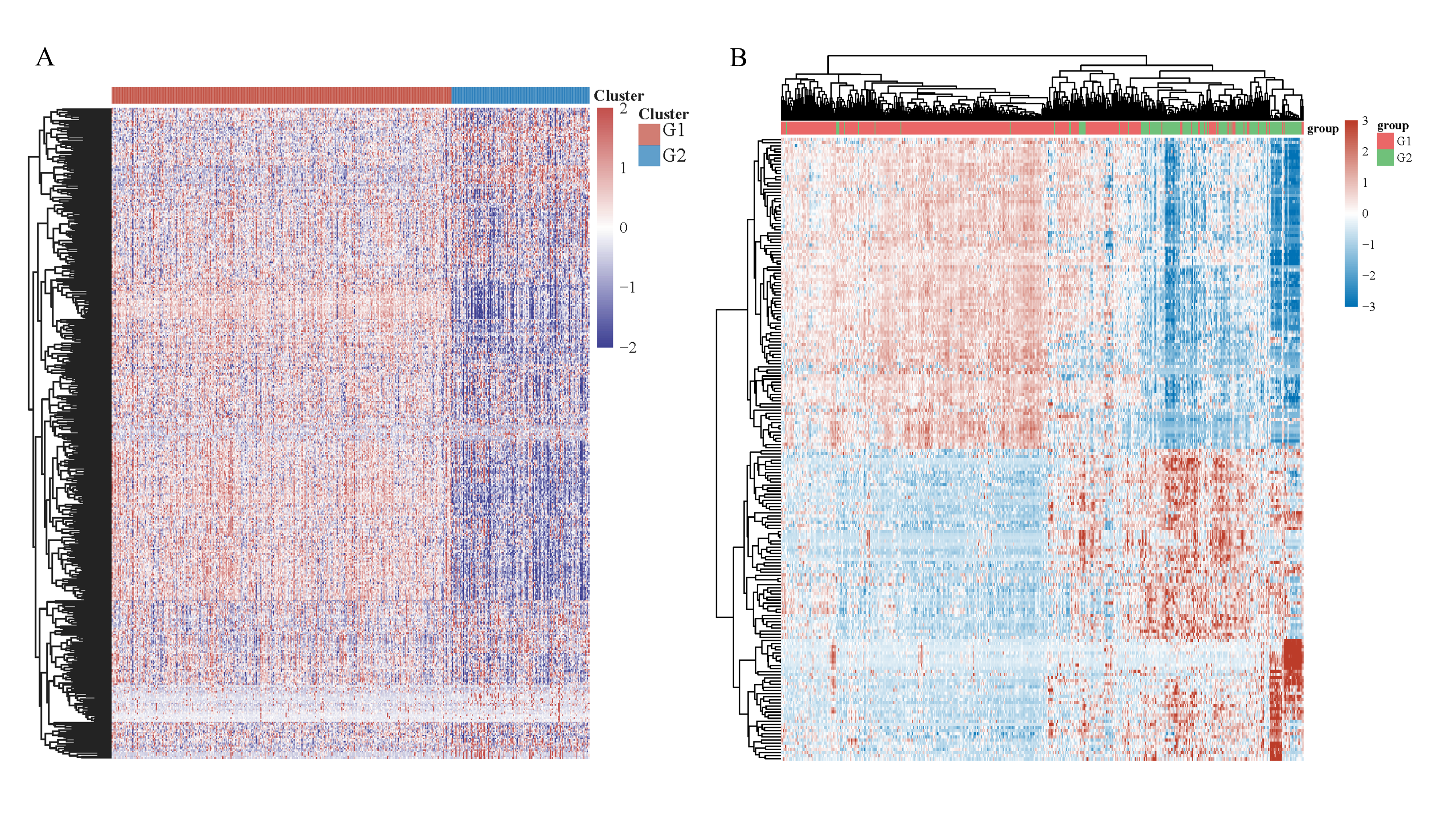

Supplement: Supplementary material 3 — Consensus clustering matrix of 530 samples from TCGA dataset for k =3 (A), 4 (B), 5 (C), and 6 (D). [file Image_3.tif]

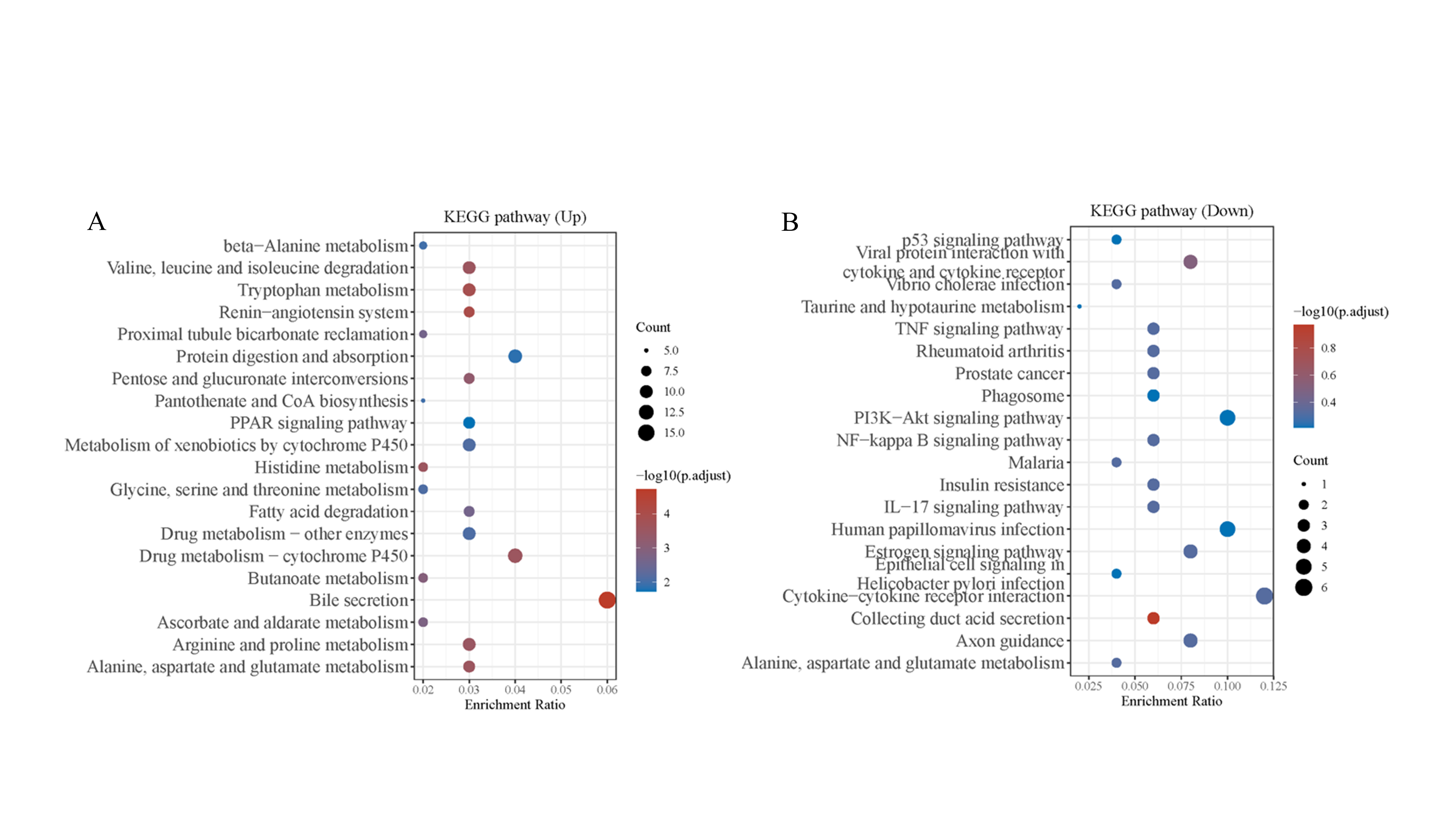

Supplement: Supplementary material 4 — (A) Heatmap of amino acid metabolism-associated gene expression in different clusters. Red represents high gene expression, and blue represents low expression. (B) Heatmap of the top 50 up- and downregulated genes with the most differential changes in two clusters. [file Image_4.tif]

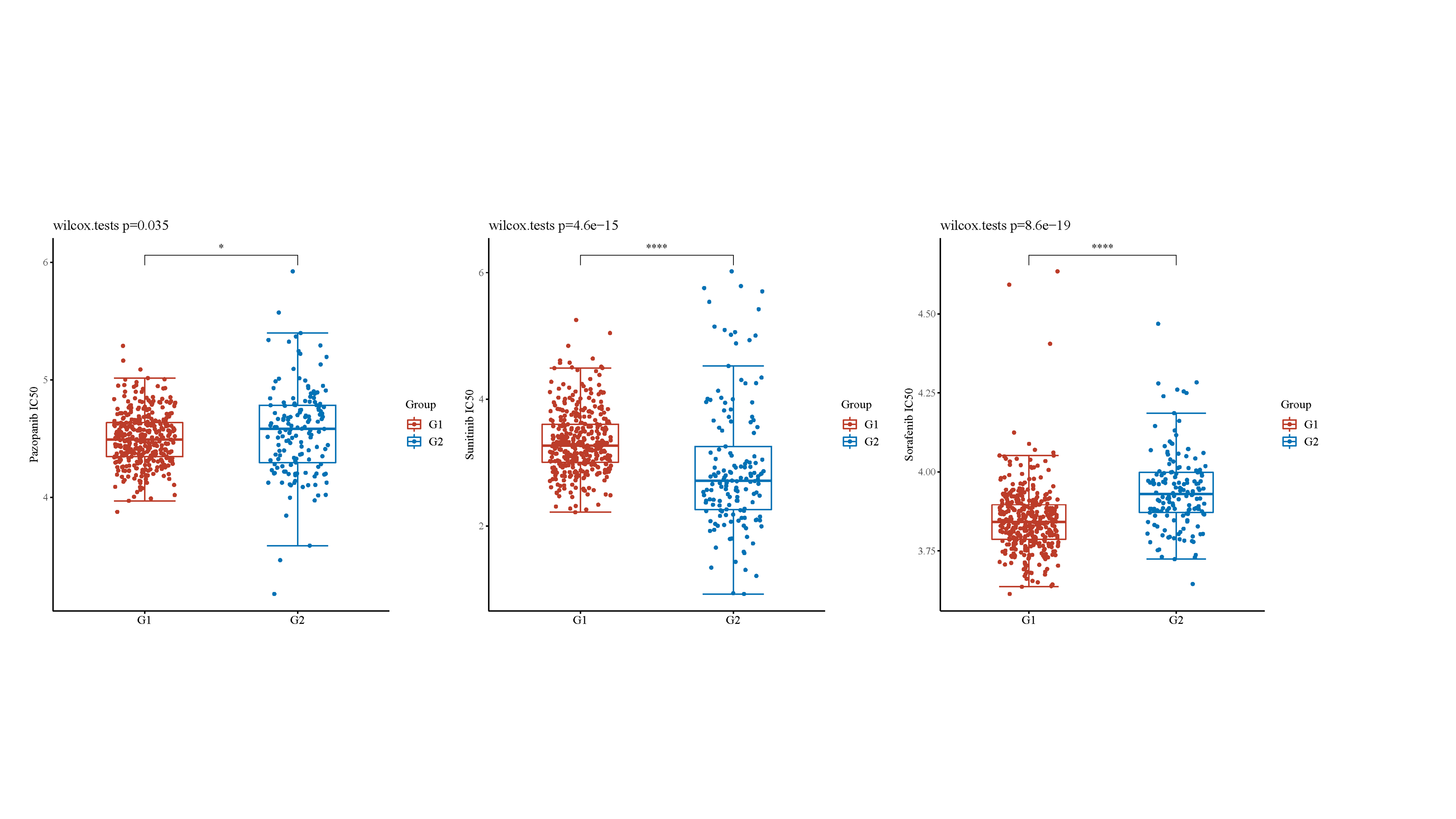

Supplement: Supplementary material 5 — KEGG analysis of DEGs that were up- (A) and down- (B) regulated in cluster 1. KEGG: Kyoto Encyclopedia of Genes and Genomes. DEG: differentially expressed genes. [file Image_5.tif]

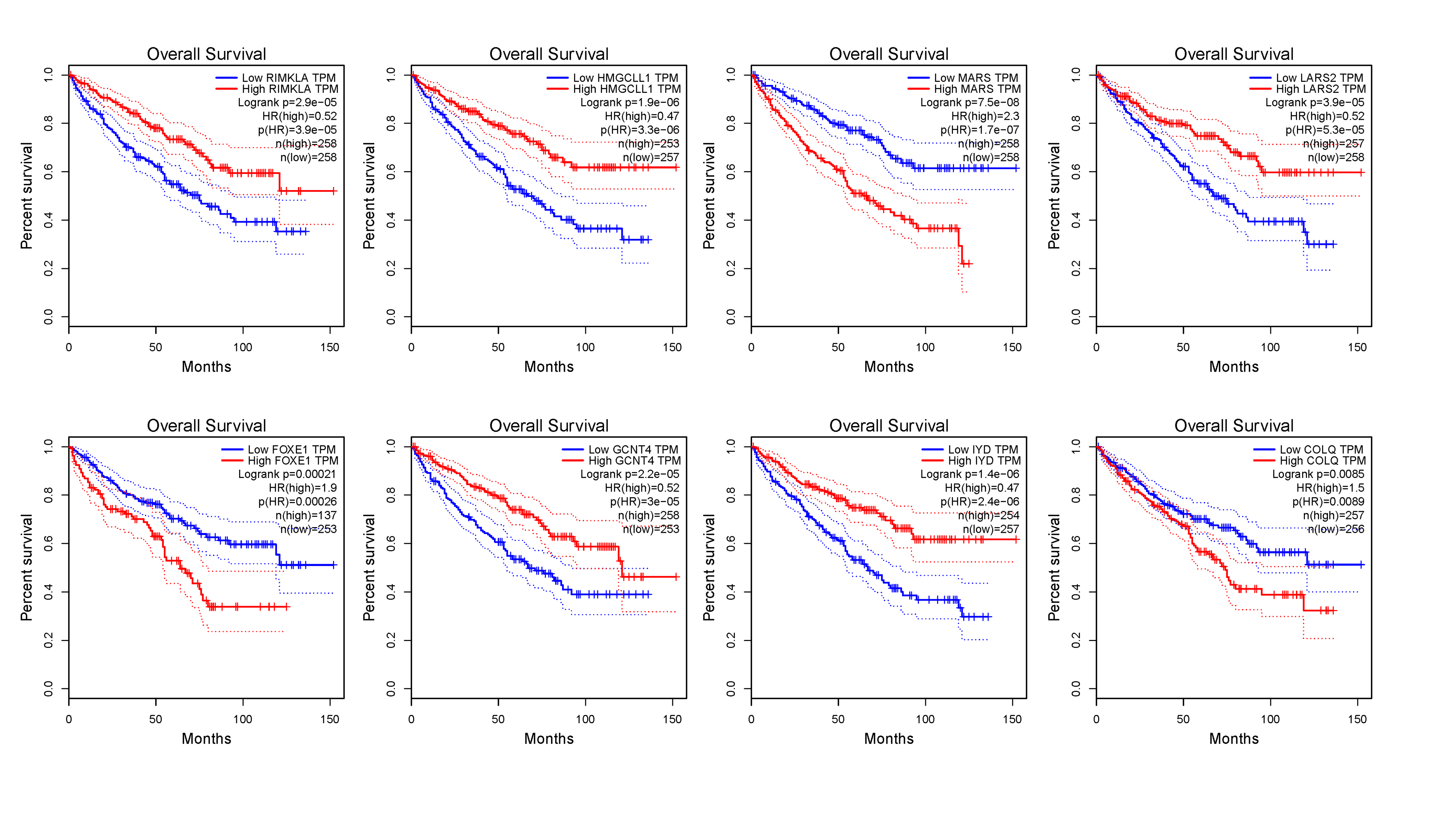

Supplement: Supplementary material 6 — Chemotherapy resistance to pazopanib, sunitinib and sorafenib of the two clusters. *p < 0.05, **p < 0.01, ***p < 0.001. [file Image_6.tif]
